# RadD Contributes to R-Loop Avoidance in Sub-MIC Tobramycin

**DOI:** 10.1128/mBio.01173-19

**Published:** 2019-07-02

**Authors:** Veronica Negro, Evelyne Krin, Sebastian Aguilar Pierlé, Thibault Chaze, Quentin Giai Gianetto, Sean P. Kennedy, Mariette Matondo, Didier Mazel, Zeynep Baharoglu

**Affiliations:** a Département Génomes et Génétique, Institut Pasteur, UMR3525, CNRS, Unité Plasticité du Génome Bactérien, Paris, France; b Sorbonne Université, Collège Doctoral, Paris, France; c Plateforme Protéomique, CNRS USR 2000, Institut Pasteur, Unité de Spectrométrie de Masse pour La Biologie, Paris, France; d Bioinformatics and Biostatistics HUB, Center of Bioinformatics, Biostatistics and Integrative Biology (C3BI), USR CNRS 3756, Institut Pasteur, Paris, France; e Center of Bioinformatics, Biostatistics and Integrative Biology (C3BI), Institut Pasteur, USR 3756 CNRS, Paris, France; UCLA School of Medicine; Northwestern University Feinberg School of Medicine; INSERM 1001; Baylor College of Medicine

**Keywords:** DNA repair, R-loop, antibiotic resistance

## Abstract

We have previously identified Vibrio cholerae mutants in which the stress response to subinhibitory concentrations of aminoglycoside is altered. One gene identified, VC1636, encodes a putative DNA/RNA helicase, recently named RadD in Escherichia coli. Here we combined extensive genetic characterization and high-throughput approaches in order to identify partners and molecular mechanisms involving RadD. We show that double-strand DNA breaks (DSBs) are formed upon subinhibitory tobramycin treatment in the absence of *radD* and *recBCD* and that formation of these DSBs can be overcome by RNase H1 overexpression. Loss of RNase H1, or of the transcription-translation coupling factor EF-P, is lethal in the *radD* deletion mutant. We propose that R-loops are formed upon sublethal aminoglycoside treatment, leading to the formation of DSBs that can be repaired by the RecBCD homologous recombination pathway, and that RadD counteracts such R-loop accumulation. We discuss how R-loops that can occur upon translation-transcription uncoupling could be the link between tobramycin treatment and DNA break formation.

## Abstract (precis)

Bacteria frequently encounter low concentrations of antibiotics. Active antibiotics are commonly detected in soil and water at concentrations much below lethal concentration. Although sub-MICs of antibiotics do not kill bacteria, they can have a major impact on bacterial populations by contributing to the development of antibiotic resistance through mutations in originally sensitive bacteria or acquisition of DNA from resistant bacteria. It was shown that concentrations as low as 100-fold below the MIC can actually lead to the selection of antibiotic-resistant cells. We seek to understand how bacterial cells react to such antibiotic concentrations using E. coli, the Gram-negative bacterial paradigm, and V. cholerae, the causative agent of cholera. Our findings shed light on the processes triggered at the DNA level by antibiotics targeting translation, how damage occurs, and what the bacterial strategies are to respond to such DNA damage.

## INTRODUCTION

Bacteria frequently encounter low concentrations (sub-MICs) of antibiotics, and recent studies point to a key role of such concentrations for the genesis of resistance mutants or exogenous resistance acquisition (1). Active antibiotics are commonly detected in soil and water. Concentrations of these antibiotics are well below the MIC but nevertheless can be found at up to several hundred nanograms/liter (2). Although sub-MICs of antibiotics do not kill bacteria, they can have a major impact on bacterial populations. In particular, it was shown that concentrations as low as 100-fold below the MIC can lead to the selection of antibiotic-resistant cells (3) through the induction of various stress responses (1, 4). SOS is one such response, triggered by a genotoxic alarm signal: single-stranded DNA, which usually results from DNA damage and/or DNA replication blockage (5). We previously found that concentrations as low as 1% of the MIC of various families of antibiotics, even those that do not cause DNA damage, such as aminoglycosides (AG), induce the SOS response in Vibrio cholerae and other pathogenic Gram-negative bacteria from different genera (6, 7). Notably, they also increase the mutation frequency and activate the oxidative stress and the RpoS general stress response pathways in both V. cholerae and Escherichia coli, which can lead to antibiotic resistance (6, 8). Reactive oxygen species (ROS) production was also shown to be central and ultimately to lead to replication and transcription stalling, triggering the SOS pathway (6, 9, 10). Aminoglycosides (such as tobramycin [TOB]) are bactericidal antibiotics that target the ribosome and prevent translation. Sub-MIC aminoglycosides nevertheless trigger the formation of DNA damage, evidenced by induction of SOS (6, 7). A genetic screen developed in our laboratory led to the identification of V. cholerae mutants in which the induction of SOS by aminoglycosides is altered (9). A number of the identified genes are involved in replication, recombination, and repair functions, suggesting that sublethal antibiotic stress is sufficient to interfere with the DNA repair and replication machineries and with RNA metabolism. Interestingly, our screen selected for mutants inactivated for the expression of proteins known to destabilize the RNA polymerase (RNAP) complex, such as Mfd. Mfd couples transcription arrests with repair by removing stalled or backtracked RNAP at bulky lesions and recruits the nucleotide excision repair (NER) machinery in a process called transcription-coupled repair (TCR) (11, 12). Stalled elongation complexes can prevent the access of DNA repair enzymes and cause replication-transcription collision. Such complexes also promote formation of structures that constitute further impediments for replication, such as R-loops. Mfd can also dislodge RNAP that pauses at abasic sites due to, for example, base excision repair of oxidative lesions (13). This is of particular interest in the case of Mfd in the response to sub-MIC tobramycin (TOB), as sub-MIC TOB treatment favors incorporation of oxidized bases into DNA (6).

In addition to Mfd, our genetic screen identified the VC1636 gene (9), which encodes a putative DNA/RNA helicase. A homolog of VC1636 was in parallel named RadD in E. coli and was shown to carry conserved helicase and DNA binding motifs (14). The closest RadD homolog was found to be the human XPB, a superfamily 2 helicase involved in transcription-coupled repair. E. coli and V. cholerae RadD proteins are 65% similar (58% identical), including helicase domains. RadD was identified recently by Cox and collaborators in a screen for genes involved in the response to ionizing radiation (15) and was suggested to have a role in DNA double-strand break (DSB) repair in E. coli (14, 16). We have identified V. cholerae VC1636 RadD as involved in the response to sub-MIC tobramycin stress. VC1636 RadD overexpression, from a high-copy-number plasmid, was able to restore survival of UV in an otherwise UV-sensitive *mfd* mutant (9), leading to the hypothesis that RadD could have a similar function as Mfd in removing stalled RNAP. A subsequent study from the Cox laboratory showed that RadD interacts with the E. coli single-stranded DNA binding protein SSB, which stimulates the ATPase activity of RadD (17), and that RadD can bind single-stranded DNA. However, the authors observed no *in vitro* helicase activity.

Here we combined high-throughput approaches and genetic characterization of multiple mutants to address the precise role of the E. coli and V. cholerae RadD proteins. For the genetic study, we focused on E. coli, since previous studies were conducted primarily in E. coli and due to the fact that V. cholerae mutants with impaired DNA double-strand break repair had poor viability. We show that sub-MIC tobramycin treatment leads to formation of double-strand DNA breaks (DSBs) in the absence of *radD* and that RNase H1 overexpression counteracts such DSB formation. Importantly, we find that the viability of the *radD* deletion mutant strongly relies on RNase H1 function. We further show that RadD directly interacts with the homologous recombination (HR) helicase RecQ. We propose that sublethal aminoglycoside treatment leads to R-loop-dependent formation of DSBs, which can be repaired by the RecBCD homologous recombination pathway, and that RadD counteracts such R-loop accumulation.

## RESULTS

### TI-seq identifies *rnhA* inactivation as highly detrimental in V. cholerae *radD*.

In order to characterize RadD, we addressed its effect in the presence of tobramycin. We adopted a high-throughput transposon insertion sequencing (TI-seq) approach to determine which genes are important in maintaining the cell integrity in the presence of antibiotics at low doses in the *radD* strain. We chose to perform the TI-seq experiments in Vibrio cholerae, because the changes caused by sub-MIC tobramycin are more marked in this species than in E. coli (6, 18), and *radD* was identified in the response to TOB in V. cholerae (9). Large transposon inactivation libraries in V. cholerae wild-type (WT) and *radD* strains were subjected to growth for 16 generations in medium without and with TOB at 50% of the MIC (0.6 μg/ml). After sequencing, insertion detection, mapping, and counts (see Text S1 in the supplemental material), we identified genes where detected insertions had at least a 4-fold increase or decrease in the *radD* strain and not in the WT after 16 generations (Table 1). We also identified genes with differential detection of insertions at *T*_0_ in the *radD* strain (Table 2). The genes marked with an asterisk in Table 2 were subsequently deleted in our WT and (when possible) in *radD* V. cholerae strains to confirm the fitness effect revealed by TI-seq (Fig. S1).

**TABLE 1:** Genes where insertions are specifically lost or enriched in the *radD* strain after evolution in MH or TOB

Decrease or increase^*e*^ and antibiotic	Gene ID		Role	Fold change for *T*_16_ vs *T*_0_ (*P* value) for strain:			
	Locus tag	Name		MH *radD* strain^*a*^	MH WT^*b*^	TOB *radD* strain^*c*^	TOB WT^*d*^
Decrease							
No antibiotic	VC1835	*pal*	Outer membrane integrity	No reads (0)	−1.8		
	VC1837*	*tolA*	Outer membrane integrity	No reads (0)	8.3		
	VC1838	*tolR*	Outer membrane integrity	No reads (0)	5.9		
	VC1839	*tolQ*	Outer membrane integrity	No reads (0)	−1.2		
	VC2291	*ngrE*	Iron and oxidative stress	No reads (0)	6.2		
	VC2292	*ngrD*	Iron and oxidative stress	No reads (0)	1.1		
	VC2293	*ngrC*	Iron and oxidative stress	No reads (0)	1.2		
	VC2294	*nqrB*	Iron and oxidative stress	No reads (0)	5.1		
	VCA0897	*pgl*	Pentose phosphate pathway	−26.3 (0.009)	2.1		
	VCA0609		Unknown	−23.4 (1.60E−15)	2.9		
	VCA0634		Putative tRNA modification	−9.3 (0.011)	1.5		
	VC2517		Putative ABC-type transport	−6.7 (1.00E−08)	1.5		
	VC2234*	*rnhA*	RNase H1, R-loop degradation	−5.9 (0.014)	−1.7		
	VC1575		Unknown	−4.1 (4.50E−07)	−1.7		
TOB, 50% MIC	VC1948		Unknown	1.6	−1.5	−30.9 (0.001)	−1.2
	VC0678	*hlyU*	Transcriptional regulator	−2.9	−3.2	−16.8 (0.001)	1.3
	ncRNA235		Noncoding RNA	−1.3	2	−16.2 (0.006)	−1.8
	VC2392*	*mutT*	Nucleotide detoxification	1	−1.8	−13.6 (8.70E−05)	−2.4
	VC2234*	*rnhA*	R-loop degradation	−5.9	−1.7	−13.5 (0.002)	−1.6
	VCA0569	*vxrE*	Unknown	−1.8	1.8	−10.3 (1.90E−04)	1.2
	VC2718	*bioH*	Metabolism	1.9	1.2	−8.7 (1.50E−04)	−2.2
	VC1759		Prophage integrase	−1.1	1.3	−8 (0.001)	1.6
	VCA0032		Unknown	−2.8	−1.4	−5.6 (2.20E−04)	−1.5
	VCA0741		Unknown	1.8	2.2	−5 (0.008)	−1.3
	VCA0654	*scrR*	Carbohydrate metabolism	−1.6	2.1	−4.9 (5.80E−05)	−1.2
	VC0099	*glpG*	Protease	1.3	−1.6	−4.8 (3.70E−06)	−1.5
	VC1824		Carbohydrate metabolism	−1.3	−1.7	−4.5 (0.004)	−1.1
	VCA0608	*yjjG*	Nucleotide detoxification	−1.2	2.1	−4.2 (1.90E−05)	−1
	VCA0501		Unknown	1.3	1.5	−4.1 (2.60E−11)	1.9

Increase							
No antibiotic	VC0887*	*yqcC*	Pseudouridine synthase (Hyp)	8.3 (0.045)	2.9		
	VC0330	*rsd*	Putative transcription factor	6.5 (0.003)	1.1		
	VC1167	*tdk*	Pyrimidine metabolism	4.7 (7.20E−09)	2.7		
TOB, 50% MIC	VC1262		Putative methyltransferase	−1.1	1.3	6.4 (3.00E−05)	1.7
	VC1150		Unknown	2.4	1.2	4.8 (1.00E−04)	2.1

a Average insertions detected in MH *radD* strain at *T*_16_ compared to *radD* strain at *T*_0_; all numbers express fold changes.

b Insertions in MH WT at *T*_16_ compared to WT *T*_0_.

c Average insertions detected in TOB *radD* strain at *T*_16_ compared to *radD* strain at *T*_0_.

d Average insertions detected in TOB WT at *T*_16_ compared to WT *T*_0_. Genes with at least 4-fold changes are shown. Deletions for genes marked with an asterisk were constructed in V. cholerae WT and *radD* strains.

e In *radD* strain but not WT at time *T*_16_.

**TABLE 2:** Genes with differential insertions at *T*_0_ in *radD* strain

Change in no. of insertions in *radD* mutant at *T*_0_ (compared to WT) and gene type	Gene ID		Role^*e*^	Normalized reads (no. of sequenced insertions) of strain at *T*_0_		Fold change of no. of insertions in *radD* strain compared to WT, both at *T*_0_^*b*^	*P* value
	Locus tag	Name		WT^*a*^	*radD* mutant		
Decrease							
DNA/RNA metabolism	VC0108	*polA*	DNA replication/repair	109	6	**19.5**	0.019
	VC0441^*d*^	*apaH*	Purine metabolism	400	90	**4.9**	0.007
	VC2392*^c^	*mutT*	Nucleotide detoxification	387	81	**4.8**	0.049
	VC2234*	*rnhA*	RNase H1, R-loop degradation	245	55	**4.4**	0.010
Translation	VC0443*^d^	*ksgA*	rRNA modification	215	13	**16.3**	0.019
	VC2679	*rpmE*	Ribosomal protein	513	39	**13.3**	0.075
	VC0582	*rsmI*	rRNA modification	387	44	**8.7**	0.019
	VC2660*	*efp*	Translation elongation factor	222	41	**5.4**	0.019
Other	VC0556*	*gshA*	Thiol redox system	134	2	**67.0**	0.040
	VC0824	*tpx*	Thiol redox system	560	85	**6.6**	0.022
	VC2381	*btuF*	Vitamin B12 ABC transporter	330	67	**4.9**	0.016
	VC2288	*nqrM*	Energy metabolism	314	69	**4.5**	0.016
	VC0240	*rfaD*	LPS	223	4	**59.4**	0.013
	VC1215	*pgsA*	Cell membrane integrity	208	7	**30.4**	0.042
	VC2156	*nlpC*	Outer membrane integrity	414	46	**9.0**	0.022
	VC1044		Unknown	622	53	**11.7**	0.038
	VC0300		Unknown	271	16	**16.9**	0.015
	VC0911	*treA*	Trehalose metabolism	331	31	**10.8**	0.032
	VC2669		Tyrosine metabolism	462	78	**5.9**	0.000
	VC0395	*gtaB*	Carbohydrate metabolism	446	53	**8.4**	0.043
	VC0964	*crr*	Carbohydrate metabolism	341	72	**4.7**	0.004
	VC0721	*pstS*	Phosphate ABC transporter	289	71	**4.1**	0.047
	VC1802		Unknown	516	19	**27.5**	0.011
	VC1810		Unknown	508	31	**16.5**	0.040

Increase	VC2326*	*yebG*	dsDNA-binding SOS protein	19	136	*7.2*	0.05
	VCA0156	*mrpC*	Electron transport	26	159	*6.2*	0.03
	VC0718*	*rdgC*	NAP	34	199	*5.9*	0.03
	VC1693	*torC*	Energy metabolism	59	255	*4.3*	0.05

a Normalized average reads.

b Values in boldface are decreases; values in italic are increases. These numbers correspond to fold changes calculated with average insertions that included decimals. Genes with at least 4-fold differences are shown.

c Deletions for genes marked with an asterisk were constructed (when possible) in V. cholerae WT and *radD* strains. *ksgA* mutants could not be obtained.

d *ksgA* and *apaH* are in the same operon.

e Abbreviations: LPS, lipopolysaccharide; dsDNA, double-stranded DNA; NAP, nucleotide-associated protein.

10.1128/mBio.01173-19.1 TEXT S1 Supplemental figure legends and methods. Download Text S1, DOCX file, 0.02 MB. Copyright © 2019 Negro et al. 2019 Negro et al. This content is distributed under the terms of the Creative Commons Attribution 4.0 International license.

10.1128/mBio.01173-19.2 FIG S1 Validation of genes identified by TI-seq through the construction of deletion mutants and growth curves in V. cholerae. Download FIG S1, TIF file, 0.8 MB. Copyright © 2019 Negro et al. 2019 Negro et al. This content is distributed under the terms of the Creative Commons Attribution 4.0 International license.

Strikingly, the number of detected insertions in *rnhA* coding for RNase H1 at *T*_0_ decreased 4.4-fold in the *radD* mutant compared to WT (Table 2), and *rnhA* inactivation was found to be highly detrimental in the *radD* strain after 16 generations in Mueller-Hinton (MH) (loss of 5.9-fold in *radD* mutant against 1.7-fold in WT) and even more so in TOB (loss of 13.5-fold in *radD* mutant against 1.6-fold in WT). We constructed single mutants of *rnhA* in V. cholerae WT; however, despite our efforts, we could not delete *rnhA* in the V. cholerae *radD* mutant (not shown). We took advantage of a thermosensitive plasmid expressing *radD_vc_* to construct *rnhA* deletion mutants at a permissive temperature in V. cholerae WT and *radD* mutant contexts, but the double mutant strains did not grow upon loss of plasmid at a nonpermissive temperature, suggesting synthetic lethality with *radD* under these conditions (Fig. S1J). In parallel, we applied a similar strategy in E. coli using P1 transduction of *rnhA* interrupted by a resistance cassette and found that the E. coli *radD rnhA* mutant could also not be constructed at a nonpermissive temperature (Fig. S1K). These results show the importance of processing R-loops in the absence of RadD and are consistent with a role of RadD related to R-loop formation/destabilization.

### Importance of genes related to DNA metabolism in the V. cholerae *radD* mutant.

At time zero and *T*_16_, a large proportion of the genes that are specifically found to be important for the *radD* strain during antibiotic stress are involved in energy metabolism, general metabolism, and membrane integrity (Tables 1 and 2), among which are two operons that become essential in the *radD* strain (no insertions detected), the proton-motive-force-dependent *tol-pal* operon ensuring membrane integrity (19) and the *ngr* operon involved in oxidative stress (20), suggesting that the *radD* strain is more sensitive to oxidative and membrane stresses. Another category includes genes related to DNA metabolism (*polA, mutT, apaH*), suggesting the increased occurrence of DNA damage in the *radD* strain. PolI (*polA*) is a DNA polymerase responsible for stripping RNA primers during lagging-strand replication but is also pivotal in various DNA repair pathways in E. coli (21, 22). ApaH is involved in detoxification of toxic DNA bases (23) and resistance to stress (24), and MutT limits incorporation of potentially mutagenic oxidized guanine residues into DNA (25). Interestingly, *mutT* inactivation detection decreased 13.6-fold in the TI-seq experiment in *radD* TOB compared with only 2.4-fold in WT TOB. Moreover, as described above for the *rnhA radD* synthetic lethality, the *polA radD* double mutant could also not be obtained in V. cholerae using the same strategy (not shown). The identification of these genes points to amplified DNA damage in the absence of *radD* and suggests that the *radD* strain is somehow less tolerant to oxidative variations (even in the absence of TOB) and could have difficulties coping with the incorporation of modified nucleotides in DNA (or RNA) compared with the WT. These results do not exclude, however, the occurrence of such stress in the WT context upon TOB treatment, consistent with our previous results showing the importance of MutT in response to sub-MIC tobramycin in V. cholerae (6). Subsequent growth assays indeed show that deletion of *mutT* causes a slight growth defect in both WT and *radD* strains (Fig. S1M). Several gene inactivations whose detections increase in *radD* were also identified (Tables 1 and 2). We constructed simple and *radD* double deletion mutants for several such genes, i.e., YqcC, putative tRNA pseudouridine synthase; RdgC, which inhibits RecA-mediated strand exchange *in vitro* (26); and YebG, belonging to the SOS regulon and DSB processing pathways (27), but we observed no significant effect on growth in either MH or TOB (not shown), although they appear to slightly increase the MIC of TOB (Fig. S1L). Further study needs to be carried out to elucidate the interplay between RadD and these factors. Finally, since RadD was previously suggested to be involved in DSB repair, we expected to find *recB* inactivation as detrimental in the *radD* context, but the stringency of our analysis did not show the loss of detected insertions in *recB* as statistically significant. This is due to the low number of initial insertions in the *recB* gene in both WT and *radD* contexts and further decreased detections after 16 generations in TOB. However, when we specifically look at the faith of detected insertions after 16 generations in the absence of TOB, the number of reads decreases 6-fold in the *radD* mutant but not in the WT, supporting the hypothesis that DSB repair is important in the absence of *radD*.

### Coupling of transcription and translation is critical in *radD* mutant and in sub-MIC TOB.

Another category of genes whose inactivation affects growth of the *radD* strain relates to translation, particularly ribosome biogenesis and stability factors (such as KsgA) and EF-P, a translation-transcription coupling factor (Table 2). Insertion counts decreased 5.4-fold for *efp* in the *radD* mutant at time zero compared to WT. We found that the deletion of *efp* affects the growth of *radD* even in the absence of antibiotics (Fig. S1A and B), suggesting that the coupling of transcription and translation is important in this mutant. Moreover, we observe that deletion of *efp* is lethal in TOB at 50% of the MIC, even in the *radD*^+^ context, highlighting the need for translation-transcription coupling upon exposure to sub-MIC TOB (Fig. S1A to C).

### RadD directly interacts with RecQ.

In parallel, in order to identify protein partners of RadD, we performed a tandem affinity purification assay (TAP-tag [Text S1] [28, 29]), under conditions with and without antibiotic stress in V. cholerae (data not shown). Selected proteins were then tested by yeast two-hybrid assay (30), among which was RecQ helicase. RecQ, together with SSB, has been previously identified in a TAP-tag assay with E. coli RadD (17), but RecQ was suggested tp be detected because of a coassociation with SSB. We observed here strong direct interaction between V. cholerae RadD and RecQ (Fig. S2). On the other hand, no interaction was observed between RadD and the RNA polymerase subunits RpoB/C (not shown).

10.1128/mBio.01173-19.3 FIG S2 RecQ_vc_ interacts with RadD_vc_ in yeast two-hybrid assay. Download FIG S2, TIF file, 0.2 MB. Copyright © 2019 Negro et al. 2019 Negro et al. This content is distributed under the terms of the Creative Commons Attribution 4.0 International license.

### The RecBCD double-strand break homologous recombination repair pathway is important in the response to tobramycin in the absence of *radD*.

**FIG 1:**
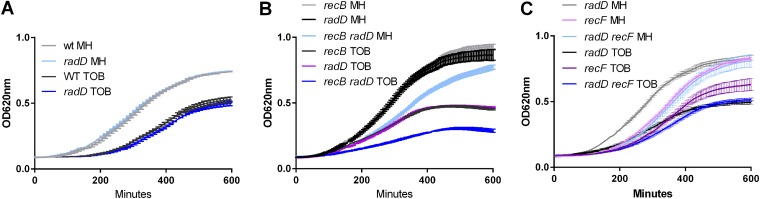
Growth of E. coli mutants in the presence of TOB at 50% of the MIC (0.25 μg/ml). Growth was measured with the Tecan Infinite plate reader. MH is rich medium without antibiotic. Each condition was tested 3 to 5 times. Standard deviations are represented. Statistical significance tests were performed on the slopes, and *P* values are represented in Table S2.

In parallel to the high-throughput approach, we undertook an extensive genetic study in E. coli, due to the fact that V. cholerae mutants with impaired DNA repair had poor viability. To analyze the response of different mutants to TOB, we assayed growth in TOB at 50% of the MIC (0.25 μg/ml for E. coli). Deletion of *radD* alone conferred no growth defect (Fig. 1A and Table S2). In order to understand which pathways could be linked with the function of RadD, we inactivated several genes related to DNA stress and repair pathways in E. coli: *recB* (HR, double-strand break repair), *recF* (HR, single-strand gap repair), *uvrA* (NER), *dinB* (translesion synthesis), and *rep* and *dinG*, which are accessory replicative helicases that clear DNA from roadblocks (31). We then tested growth of single and double E. coli mutants in MH and TOB. No negative effect was observed for deletion of *uvrA*, *dinG*, *rep*, and *dinB* in the *radD* context, in MH, or in TOB (Fig. S3), consistent with TI-seq data. This suggests that replication in the *radD* mutant is not impaired by roadblocks and bulky complexes or lesions and that NER is not needed. On the other hand, inactivation of *recB* (Fig. 1B) but not *recF* (Fig. 1C) was observed to be detrimental in the *radD* mutant. This points to DSB formation in the *radD* mutant in the presence of TOB, and even in MH without antibiotic, thus requiring RecBCD homologous recombination.

10.1128/mBio.01173-19.4 FIG S3 Growth of various E. coli repair and replication mutants in the presence of TOB at 50% of the MIC. Download FIG S3, TIF file, 0.3 MB. Copyright © 2019 Negro et al. 2019 Negro et al. This content is distributed under the terms of the Creative Commons Attribution 4.0 International license.

10.1128/mBio.01173-19.8 TABLE S2 Statistical analysis. Download Table S2, XLSX file, 0.02 MB. Copyright © 2019 Negro et al. 2019 Negro et al. This content is distributed under the terms of the Creative Commons Attribution 4.0 International license.

**FIG 2:**
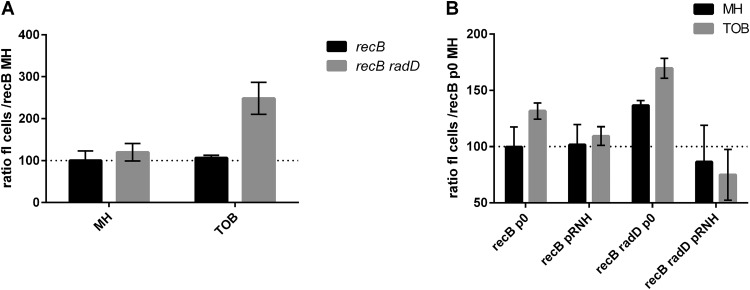
Quantification of DNA double-strand breaks in E. coli. TUNEL assays were performed (see Materials and Methods), and fluorescence was measured by flow cytometry (MACSQuant). Standard deviations are represented. Statistical significance tests (*t* tests) were performed, and *P* values are represented in Table S2. MH, no antibiotic; TOB, 0.2 μg/ml; p0, empty pTOPO vector; pRNH, pTOPO::rnhA_ec_ (plasmids pB352 and pI388 are shown in Table S1).

DSB formation in E. coli *recB* and *recB radD* strains was quantified using a fluorometric terminal deoxynucleotidyltransferase-mediated dUTP-biotin nick end labeling (TUNEL) assay. In this system, double-strand ends, including those generated by DSBs, are fluorescently labeled and quantified by flow cytometry. We used *recB* derivatives for this assay, so that DSBs that are formed cannot be repaired and thus can be accurately measured. Figure 2A (and Table S2 for statistical significance) shows that fluorescence is increased in the *recB radD* strain compared to the *recB* single mutant in TOB. These results are consistent with the hypothesis that DSBs are formed in the *radD* mutant in sub-MIC TOB and that these breaks are repaired by the RecBCD HR pathway.

10.1128/mBio.01173-19.7 TABLE S1 Strains, plasmids, and oligonucleotides. Download Table S1, XLSX file, 0.03 MB. Copyright © 2019 Negro et al. 2019 Negro et al. This content is distributed under the terms of the Creative Commons Attribution 4.0 International license.

### R-loops are responsible for part of the DSBs formed in the absence of *radD* in TOB.

RadD had been reported previously to be involved in DSB repair (14, 16), but no molecular mechanism was proposed. Having identified *radD* in a stalled-transcription screen (9), and based on our TI-seq data identifying *rnhA* deletion as detrimental in the *radD* mutant, we were in a position to ask the question of whether DSBs formed in the *radD* mutant could arise from R-loops. Indeed, R-loops are frequently formed under conditions where RNAP stalls (32, 33) and can be at the origin of DSB formation when they are not degraded by RNase H1 (*rnhA*) (32, 34).

**FIG 3:**
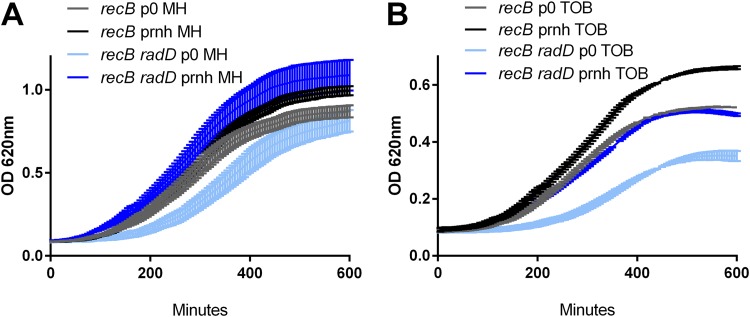
Effect of RNase H overexpression on growth of E. coli mutants in *recB*-deficient context. Growth was measured with the Tecan Infinite plate reader. MH is rich medium without antibiotic. An 0.2-μg/ml concentration of TOB was used in the *recB*-deficient context (instead of 0.25 μg/ml) because of decreased viability of *recB* mutants. Each condition was tested at least 3 times. Standard deviations are represented. Statistical significance tests were performed on the slopes, and *P* values are represented in Table S2. Plasmids are as in Fig. 2.

In order to test this hypothesis, we first undertook the construction of various *rnhA* mutant derivatives in E. coli. However, as previously described (33, 35–38), all the strains carrying *rnhA* inactivation quickly accumulated suppressor mutations. A second strategy was used to look for the phenotype of RNase H1 overexpression in our different mutants: we compared growth of E. coli *recB* and *recB radD* strains transformed with a plasmid overexpressing RNase H1 or with an empty plasmid. We also tested isogenic *recB*^+^ strains. No effect of RNase H overexpression was observed in the *recB*^+^ context for the WT and *radD* mutant (Fig. S4). In the *recB*-deficient context, although we observed a slight improvement by RNase H1 overexpression on growth of the *recB* strain in TOB (Fig. 3A), the effect was even more marked in the *recB radD* mutant, where pRnhA^+^ significantly improved growth (Fig. 3B).

10.1128/mBio.01173-19.5 FIG S4 Effect of RNase H1 overexpression on growth of E. coli mutants in *recB*^+^-proficient context. Download FIG S4, TIF file, 0.1 MB. Copyright © 2019 Negro et al. 2019 Negro et al. This content is distributed under the terms of the Creative Commons Attribution 4.0 International license.

We quantified DSB formation in the presence of the RNase H1-overexpressing plasmid in E. coli (Fig. 2B). Interestingly, introduction of the empty plasmid led to slightly higher DSB levels in the *recB* mutant in TOB. In the *recB radD* context with empty plasmid, DSB levels were increased compared to the *recB* strain, which was consistent with what was observed in the plasmidless assay (Fig. 2A). When pRnhA^+^ was introduced in the *recB radD* context, the DSB levels decreased compared to the isogenic strain with empty plasmid in TOB. These results suggest that the overexpression of RNase H1 relieves DSBs that are formed in the absence of *radD* in TOB and that R-loop formation at least partly accounts for the viability loss of the *radD* mutant, suggesting that *radD* could have a role in the avoidance/destabilization of R-loops.

### RadD is involved in R-loop degradation/limitation *in vivo*.

**FIG 4:**
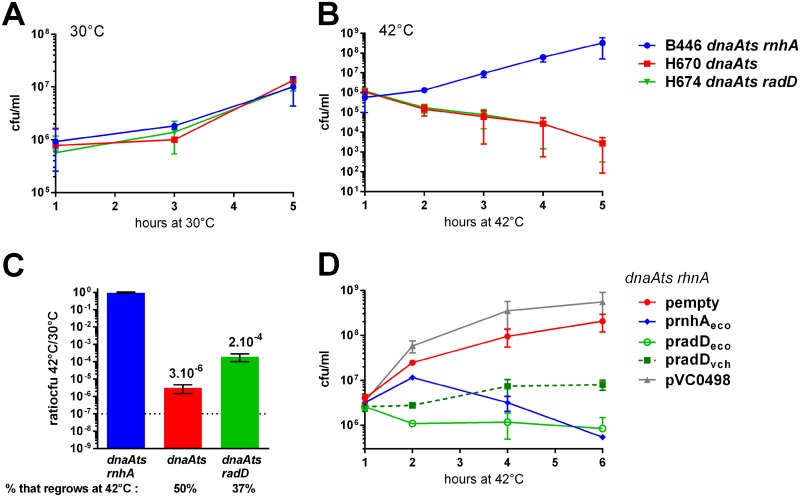
Effect of RadD in R-loop-dependent stable DNA replication in E. coli *dnaA*(Ts) mutant. Cultures were started at 30°C and were kept at 30°C (permissive) or shifted at 42°C (nonpermissive temperature) at time zero. (A, B, and D) Numbers of CFU are represented over time after time zero (hours). When a plasmid was present, carbenicillin (100 μg/ml) was added to the medium. (C) Overnight cultures were plated at 30°C and 42°C, and the ratios of CFU are shown. pempty, empty pTOPO vector; prnhA_eco_, pradD_eco_, pradD_vch_, and pVC0498, plasmids expressing the corresponding genes (plasmids pB352, I388, I605, I468, and I391 are shown in Table S1).

We were unable to test direct unwinding of RNA-DNA hybrids *in vitro* despite our efforts to purify an active form of V. cholerae RadD. In order to address whether R-loop formation is increased *in vivo* in the absence of RadD, we used the properties of the *dnaA*(Ts) *rnhA* mutant where stable DNA replication occurs at R-loops throughout the chromosome (39). As DnaA is essential for priming of chromosome replication in E. coli, the *dnaA*(Ts) thermosensitive mutant cannot grow at 42°C. Inactivation of RNase H1 (*rnhA*) in the *dnaA*(Ts) mutant restores viability because of increased formation of R-loops, which can prime replication initiation. We hypothesized that if R-loop formation is increased in the *radD* mutant, then the *dnaA*(Ts) *radD* strain would also grow at 42°C. Figure 4 shows that although all mutants have similar growth profiles at the permissive temperature (30°C, Fig. 4A), only the inactivation of *rnhA* restores viability at 42°C, and not *radD* (Fig. 4B). This means that the number of R-loops that are formed upon *radD* deletion is not increased to levels sufficient to promote stable replication in the *dnaA*(Ts) background. However, when these mutant strains were grown at 30°C and then restreaked at 42°C, we observed growth of several colonies in the *dnaA*(Ts) *radD* strain but not in the *dnaA*(Ts) strain. We quantified the appearance of these colonies by plating the cultures at 42°C and observed that there is an increase of CFU from 3 × 10^−6^ in the *dnaA*(Ts) strain to 2 × 10^−4^ in the *dnaA*(Ts) *radD* strain (Fig. 4C). Since spontaneous mutation frequencies were not increased in the *radD* or *radD dnaA*(Ts) strain compared to isogenic *radD*^+^ strains (data not shown), this ∼100-fold increase of spontaneously growing colonies was unexpected. These CFU could appear due to genetic suppression mutations or to stochastic phenotypic variation. When we restreaked these CFU at 42°C, only 37% to 50% grew again, independently of the fact that the strain was deleted or not for *radD* (Fig. 4C). These results suggest that more R-loops are formed stochastically in the *radD* strain and that this phenotype cannot be inherited, meaning that at least half of the obtained CFU are not genetic suppressors. A major difference between planktonic and colony growth is oxygen availability. One possible explanation for the growth of the *radD dnaA*(Ts) strain in solid and not liquid medium could be that the strain could be particularly sensitive to oxygen and therefore will grow only in colonies that are under mostly anaerobic growth. Using a high-copy-number plasmid (∼100 copies), we next addressed whether overexpression of RadD has a negative effect on R-loop formation. The *dnaA*(Ts) *rnhA* strain with empty plasmid grows at 42°C (Fig. 4D). Reintroduction of *rnhA* in *trans* prevents growth as expected, and so does overexpression of E. coli *radD* and V. cholerae *radD*. In order to ascertain that growth prevention is not due to protein overexpression, we also expressed a V. cholerae protein with a putative RNase function (VCA498) and found no effect on growth. Altogether, these results show that RadD overexpression has a negative effect on R-loop formation. R-loop formation is under some conditions related to DNA superhelicity levels. We tested *in vivo* whether supercoiling levels could be different in the *radD* mutant using an assay developed previously in the laboratory (40) and found that RadD has an impact on DNA topology (Fig. S5); however, chloroquine gels to test plasmid supercoiling in the presence or absence of RadD did not yield conclusive results regarding an effect of RadD on topology in this assay (not shown).

10.1128/mBio.01173-19.6 FIG S5 Impact of RadD in cruciform extrusion during integron recombination. Download FIG S5, TIF file, 0.1 MB. Copyright © 2019 Negro et al. 2019 Negro et al. This content is distributed under the terms of the Creative Commons Attribution 4.0 International license.

## DISCUSSION

We show here that in the absence of *radD*, V. cholerae relies on various factors, such as RNase H1, for efficient response to sub-MIC TOB. The results also highlight the fact that the presence of sub-MIC TOB leads to DSBs, at least partly through R-loop formation, explaining the need for DSB repair in the absence of RadD.

In previous studies, the E. coli *radD* single mutant showed only a very small defect in survival of UV irradiation compared to the WT strain (14), unlike the UV-sensitive *mfd* mutant (41). When we further addressed the role of RadD in the response to UV damage, and a possible link with Mfd, the *radD mfd* double mutant showed higher UV sensitivity than the *mfd* single mutant, suggesting that RadD and Mfd may have overlapping functions in response to UV irradiation (not shown). The absence of these factors affects also the response to sub-MIC TOB, pointing to impaired transcription.

The link between transcription impediments, R-loop formation, and DSBs has been described in prokaryotes and eukaryotes. It is known that R-loops accumulate at stalled transcription elongation complexes (32, 33) and in the absence of effective transcription termination (42). In human cells, it was shown that R-loops provoke DSB formation by interfering with replication (43–45). In bacteria, replication-transcription collisions are known to lead to genomic instability and breaks (32, 46). Previous work has established that R-loops generate DSBs because they constitute replication blocks and that RNAP backtracking is an important factor potentiating the formation of such R-loop extensions and DSBs (32). The fact that we did not see any effect of the inactivation of replicative helicases such as Rep or DinG in the *radD* strain suggests that the absence of *radD* does not cause replication blocks. However, R-loop-dependent genome instability is not necessarily due to replication blocks. In a recent study, it was shown that R-loop-dependent DSB formation in E. coli was due not to replication impairment but to formation of RNA gaps at R-loops (RNA-DNA junctions at arrays of R-loops), which lead to chromosomal DSBs (34). Importantly, overexpression of RNase H1 and active antibacktracking mechanisms suppress such DSB accumulation in E. coli (32). Another HR helicase proposed to prevent R-loop formation is RecG (47). Deletion of *recG* is colethal with *rnhA* and promotes stable DNA replication. However, our TI-seq data predict no colethality of *recG* and *radD*, as insertions in *recG* are detected at equivalent levels in WT and *radD* strains in the presence or absence of TOB. On the other hand, the E. coli *radD recG* mutant was previously studied (14) and the authors found that the strain rapidly accumulates suppressors and proposed that this could be due to a DSB repair defect. Here, RecG does not appear to be important in the absence of RadD, but one cannot rule out the possibility that RecG and RadD may have overlapping functions against R-loops. DSB formation could also be linked to DNA structures formed upon inappropriate R-loop processing in a *radD* mutant. R-loops can also interfere with DNA damage repair. It was shown in yeast that RNase H1 is important at DSBs against R-loops which otherwise impair recruitment of RPA (replication protein A, the SSB orthologue; SSB) and subsequent access of HR proteins to DSBs (48). Along the same line, a recent study showed that the human transcription-coupled repair protein CSB is recruited to R-loops induced by reactive oxygen species (ROS) at transcribed sites to initiate repair by HR (49).

How RadD counteracts/reduces R-loop formation is unclear. One possibility is through an effect on DNA supercoiling, which is linked to R-loop formation. In E. coli, TopoI is known to interact with RNAP and reduce R-loops (50) and its depletion leads to negative supercoiling behind the transcribing RNAP, enhancing R-loop formation (36). We observed that RadD has an impact on DNA topology, but this effect can also be indirect.

On the other hand, we know that RadD interacts with SSB (17) and with RecQ (this study). One possible hypothesis for RadD action would thus be that RadD together with RecQ could directly destabilize/unwind R-loops and recruit SSB at DSBs. SSB stimulates the activity of RNase H1 (51) and enhances the DNA helicase activity of RecQ in E. coli (52) and human cells (53, 54). RecQ can impact R-loop formation (55, 56) through effects on replisome stability at transcription-replication conflicts or direct unwinding of R-loops (57) or through changes in superhelicity (57–59). *topA* and *recQ* mutant backgrounds could be used in future work to more clearly define the role of RadD.

Interestingly, it was shown that the eukaryotic RecQ5 associates with RNAP and enforces the stability of ribosomal DNA arrays (60). Translating ribosomes also inhibit DSB formation at transcription sites (33). Indeed, slowing or blocking translation leads to DSB formation in the absence of R-loop repair (34). Thus, a role for RadD-RecQ can also be envisaged at the translation-transcription level. We can speculate that RadD could be important under conditions where translation is slow/impaired for the following reasons: (i) RadD is involved in the response to TOB, which interferes with translation; (ii) slow translation can promote R-loop formation; and (iii) our TI-seq experiment identified several translation-related factors that are important for the fitness of the *radD* mutant (Tables 1 and 2), namely, EF-P and KsgA. KsgA is a ribosome biogenesis and stability factor. EF-P counteracts ribosome pausing and maintains transcription-translation coupling (61).

Coupling of transcription and translation reduces R-loop formation in bacteria and subsequent DSB formation, as a newly transcribed RNA can be bound immediately by ribosomes (62). In E. coli, RNA polymerase also directly binds to ribosomal subunits *in vivo*, which could facilitate coordination of transcription and translation (63). In fact, the rate of transcription was shown to be controlled by the rate of translation (64). Slow translation leads to RNAP backtracking (65, 66). Accordingly, translation prevents transcription-related formation of DSBs (32). Transcription-translation coupling can be disrupted upon ribosome stalling (in the *efp* mutant or when aminoacyl-tRNAs are limiting [67, 68]). Notably, the EF-P transcription-translation coupling factor was identified as a suppressor of the growth defect in the *rnhA topA* mutant (69), suggesting that translation can also counteract R-loops that are formed due to accumulated negative supercoiling. Another example is the *rep uvrD* mutant, which is lethal due to conflicts between replication and transcription elongation complexes. This lethality can be suppressed by rpo* alleles destabilizing RNAP (31) but also by mutations in EF-P (70). One hypothesis regarding the anti-R-loop action of RadD could therefore be at the level of translation-transcription coupling. Under this model, the involvement of RadD in the response to TOB effects of ribosome progression is coherent.

**FIG 5:**
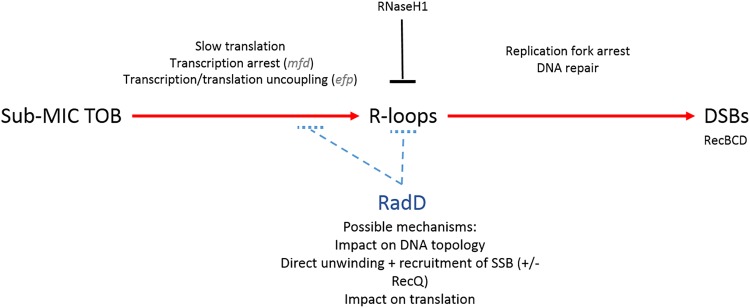
RadD counteracts formation of DSBs arising from R-loops. We propose that sub-MIC TOB impedes translation, leading to transcription defects, thus enhancing R-loops/R-lesions at transcription sites, causing DSBs that are repaired by the RecBCD pathway. We hypothesize that RadD (possibly with RecQ) acts either at the level of translation-transcription coupling for the avoidance of R-loop formation or directly at the R-loop before DSBs arise. Shown in parentheses are genes that are mentioned in the text and steps where they could be involved.

Here, we have initially addressed the function of RadD in response to sub-MIC tobramycin. In the light of our results and the discussion above, we propose that TOB, even at sub-MIC levels, impedes translation, which primes defects in transcription, thus enhancing R-loops/R-lesions at transcription sites, causing DSBs that are repaired by the RecBCD HR pathway. SOS is indeed triggered here by DSB repair as observed previously (7, 9). We hypothesize that RadD, together with RecQ, acts either at the level of translation-transcription coupling for the avoidance of R-loop formation or directly at the R-loop before DSBs arise (Fig. 5). Further study is needed to unravel the exact mechanism of action of RadD on R-loops. Interestingly, the *radD* gene is located next to the *rsuA* gene putatively involved in ribosome assembly. Although we found no direct interaction between the RadD and RsuA proteins (two-hybrid data, not shown), we observe that the synteny is conserved among many gammaproteobacterial genera, such as *Escherichia*, *Klebsiella*, *Salmonella*, *Serratia*, and *Shewanella*. Finally, sub-MIC TOB may not affect all ribosomes equally, leading to heterogeneity of responses within a clonal population. Single-cell approaches (such as microfluidics) would be complementary and suitable in future research to compare behaviors and responses at both subpopulation and whole-population levels.

## MATERIALS AND METHODS

MH medium was used for the study of the effect of sub-MIC tobramycin. TOB was aliquoted and stored at a 10-mg/ml concentration at −20°C. A fresh aliquot was used for each experiment.

Plasmids, strains, and oligonucleotides used in this study and their constructions are listed in Table S1 in the supplemental material. E. coli mutants were constructed by P1 transduction, and V. cholerae mutants were constructed by homologous recombination after natural transformation or with a conjugative suicide plasmid (pMP7 = pWS7848) as described previously (6, 71, 72).

Growth kinetics were performed from overnight cultures from single colonies, using the Tecan Infinite plate reader on 96-well plates for 10 h at 37°C with shaking. OD_620_ was measured every 5 min.

Growth curves (CFU counts) of the *dnaA*(Ts) derivatives were performed as previously described (9).

Double-strand break quantification was performed using the Promega fluorometric TUNEL system. An overnight culture was diluted 100× in MH with or without 0.2 μg/ml TOB and grown to an OD_620_ of 1. Carbenicillin (100 μg/ml) was added to the growth medium for plasmid-carrying strains. One milliliter (3 × 10^6^ to 5 × 10^6^ cells) was centrifuged at 2,000 rpm for 15 min at 4°C, washed twice with cold PBS, and resuspended in 500 μl PBS. Cells were fixed with 5 ml 1% methanol-free formaldehyde on ice for 20 min, washed twice with cold PBS, and permeabilized overnight with 5 ml ice-cold 70% ethanol. Cells were then washed twice with PBS and stained according to the manufacturer’s recommendations. Green fluorescence was measured on a Miltenyi MACSQuant flow cytometer.

Transposon insertion sequencing libraries were prepared as previously described (9, 73) to achieve a library size of 600,000 clones and subjected to passaging in MH and MH with TOB at 0.5 μg/ml for 16 generations. Sequencing and analysis are described in detail in the supplemental material (see Text S1). Briefly, sequencing libraries were prepared using Agilent’s Sureselect XT2 kit with custom RNA baits designed to hybridize the extremities of the Mariner transposon. Illumina paired-end sequencing technology was used, producing 2- by 125-bp-long reads. Reads were filtered through transposon mapping to ensure the presence of an informative transposon/genome junction as described previously (74). Expansion or decrease of fitness of mutants was calculated in fold change with normalized insertion numbers (75). Baggerly’s test on proportions (76) was used to determine statistical significance, and Bonferroni correction was applied for multiple testing.

### Accession number(s).

Accession numbers for the TI-seq reads are SRR8361877, SRR8361874, SRR8361875, SRR8361872, SRR8361873, SRR8361870, SRR8361871, SRR8361878, and SRR8361876 for the *radD* strain and SRR8351961, SRR8351962, SRR8351957, SRR8351958, SRR8351959, SRR8351960, SRR8351965, SRR8351966, SRR8351963, SRR8351964, SRR8351967, and SRR8351968 for the WT strain.
