# Supplementary material for: RadD Contributes to R-Loop Avoidance in Sub-MIC Tobramycin
Source: mBio. 2019 Jul 2;10(4):e01173-19. doi: 10.1128/mBio.01173-19 (PMC6606805; doi:10.1128/mBio.01173-19)
Supplement: TEXT S1 [file mBio.01173-19-s0001.docx]

**Text S1**

**Supplementary figure legends**

**Fig. S1**. **Validation of genes identified by TI-seq through the construction of deletion mutants and growth curves in *V. cholerae*.**  **A to I** (*V. cholerae*): Growth was measured with the TECAN infinite. TOB was used at 0.6µg/ml (50% MIC). Each strain was tested at least 3 times. Standard deviations are represented. ***polA*** mutant could be constructed in WT but could not be obtained in *radD,* suggesting synthetic lethality. ***ksgA*** mutants could not be constructed in any context, suggesting essentiality in our growth conditions. **J**: synthetic lethality of *rnhA* and *radD* in *V. cholerae*. ***rnhA*** mutants were constructed by conjugation and allelic replacement (see Methods) at 30°C in WT and *radD* strains carrying a plasmid pGBts-radD+ with conditional replication origin that cannot replicate at 42°C. 4 colonies were streaked at 30°C and 42°C for WT and *radD rnhA* pGBts-radD+. *radD rnhA* cannot grow upon loss of plasmid expressing RadD. **K:** synthetic lethality of *rnhA* and *radD* in *E. coli*. *rnhA*::spec and *lacZ*::cm (control) deletion alleles were P1 transduced in parallel into *E. coli* WT and *radD* strains carrying pGBts-*radD*+, expression was allowed at 30°C for 2 hours before plating at 42°C, on selective plates with and without TOB 50% MIC (0.25µg/ml). No *rnhA* transductant could be obtained in *radD* context. *rnhA* deletion mutants were also constructed in the absence of the complementation plasmid, by suicide conjugation and allelic replacement (see Methods), replacement efficiency was 3.6.10^-4^ in the WT context, and 7.8.10^-6^ in the *radD* context, which corresponds to the spontaneous mutation frequency of *V. cholerae*, suggesting that such *rnhA* deletion mutants obtained in the *radD* context probably bear suppression mutations. **L**: *V. cholerae* ***yebG***, ***rdgC***, ***yqcC*** deletion mutants where insertions were enriched in TI-seq data (positive effect of inactivation) were constructed, and MICs were measured on overnight cultures using e-tests (Biomérieux). The mutants had no significant effect on growth neither in MH nor TOB (not shown)***.***

**Fig.S2** **RecQ_vc_ interacts with RadD_vc_ in yeast two hybrid assay***. V. cholerae* RecQ/RadD interaction was tested by yeast two hybrid assay. We used as positive control *V. cholerae* Mfd and RpoB as they were shown to interact directly [1]. We observe strong interaction between *V. cholerae* RadD and RecQ. RecQ is a helicase involved in the RecFOR pathway of homologous recombination. Our TI-seq results show that RecFOR is not required for growth in the absence of RadD. Consistently, we saw no difference in growth between *recQ* and *radD recQ* mutants in *E. coli* and *V. cholerae* (not shown). – LW: diploid strains carrying both prey and bait plasmids grow in drop out medium without leucine and tryptophan. –LWH X-gal: upon interaction of bait and prey, diploid strains carrying both plasmids grow in the absence of histidine and synthesize LacZ. 1mM aminotriazole was used to select for specific interactions. p0: empty plasmid.

**Fig. S3**. **Growth of various *E. coli* repair and replication mutants in the presence of TOB at 50% of the MIC** (0.25 µg/ml). Growth was measured with the TECAN infinite. MH is rich medium without antibiotic. Each condition was tested at least 3 times. Standard deviations are represented.

**Fig S4.** **Effect of RNaseH1 overexpression on growth of *E. coli* mutants in *recB+* proficient context**. Growth was measured with the TECAN infinite. MH is rich medium without antibiotic. Each condition was tested at least 3 times. Standard deviations are represented.

**Fig. S5. Impact of RadD in cruciform extrusion during integron recombination.** In order to test whether RadD has an impact on DNA superhelicity levels, we took advantage of the properties of the integron integrase recombination. Briefly, the integration of an integron cassette by site specific recombination requires the extrusion of a cruciform structure from dsDNA. This recombination occurs between recombination sites called *attC* (where the cruciform extrudes) and *attI* (where DNA remains double stranded). This is facilitated by DNA supercoiling, and, in topoisomerase and gyrase mutants, the recombination efficiency is significantly lower (ref [46] in manuscript). We measured the recombination frequency in WT, *topA gyrB*, *radD* and *radD topA gyrB* mutants, in order to address whether RadD is involved in DNA supercoiling. As observed previously, the *topA gyrB* strain has lower *attC* x *attI* recombination frequency. Interestingly, deletion of *radD* also reduces the *attC* x *attI* recombination frequency, whereas deletion of *mfd* used as negative control here has no effect. We also tested the *attI* x *attI* recombination where DNA topology has no influence on recombination frequency (no cruciform needed), and we observed no effect of deletion of *radD*. These results suggest that RadD may have an impact on DNA topology, observed here at DNA cruciform extrusion sites.

**Supplementary methods**

**Transposon insertion sequencing (TI-seq).** A saturated mariner mutant library was generated by conjugation of plasmid pSC1819 from *E .coli* to *V. cholerae* WT and *ΔradD* as previously described [8]. Briefly, pSC189 [8, 20] was delivered from *E. coli* strain 7257 (β2163 pSC189::spec, laboratory collection) into the *V. cholerae* F606 strain and the *ΔradD* G927 strain. Conjugation was performed for 2 h on 0.45 µM filters. The filter was resuspended in 2 ml of MH broth. Petri dishes containing 100 µg/ml spectinomycin were then spread. The colonies were scraped and resuspended in 2 ml of MH. When sufficient single mutants were obtained (>600 000 for 6X coverage of non essential regions), a portion of the library was used for gDNA extraction using Qiagen DNeasy® Blood & Tissue Kit as per manufacturer’s instructions. This was used for library validation through insert amplification by nested PCR using a degenerate primer (ARB6), which contains 20 defined nucleotides followed by a randomized sequence. This was combined with a primer anchored in the edge of the transposon sequence (MV288). After this, primer ARB3, which contains the first 20 nucleotides of ARB6 was used for nested amplification in combination with MV288. After validation, the libraries were passaged in MH media for 16 generations with or without 50% of the tobramycin, in triplicate. gDNA from time point 0 and both conditions after 16 generation passage in triplicate was extracted. Sequencing libraries were prepared using Agilent’s sureselect XT2 Kit with custom RNA baits designed to hybridize the edges of the Mariner transposon. The 100 ng protocol was followed as per manufacturer’s instructions. A total of 12 cycles were used for library amplification. Agilent’s 2100 bioanalzyer was used to verify the size of the pooled libraries and their concentration. HiSeq Paired-end Illumina sequencing technology was used producing 2x125bp long reads. Reads were then filtered through transposon mapping to ensure the presence of an informative transposon/genome junction using a previously described mapping algorithm [21]. Informative reads were extracted and mapped. Reads were counted when the junction was reported as mapped inside the ORF of a gene plus an additional 50 bp upstream and downstream. Normalization calculations were applied according to van Opijnen et al [22]. Expansion or decrease of fitness of mutants was calculated in fold changes with normalized insertion numbers. Baggerly’s test on proportions [23] was used to determine statistical significance as well as a Bonferroni correction.

**Tandem affinity purification assay**. *V. cholerae* strains carrying C-ter tagged RadD and control with tag only were grown in MH and MH containing sub-MIC TOB (0.6µg/ml) or CIP (0.002 µg/ml) in triplicates in 1L cultures to OD 0.8. Cell extract were harvested as described [50, 51]. Samples were then analyzed by mass spectrometry.

**Mass spectrometry analysis**. Protein digestion. Protein samples were solubilized in urea 8 M, Tris 100 mM pH8.0, disulfide bonds were then reduced with 5 mM TCEP for 30 min at 23°C and alkylated with 20 mM iodoacetamide for 30 min at room temperature in the dark. Subsequently, LysC (Promega) was added for the first digestion step (500 ng) for 3h at 30°C. Then the sample was diluted down to 1 M urea with 50 mM Tris pH 8.0, and trypsin (Promega) was added to the sample (800 ng for 10h at 37°C). Digestion was stopped by adding 1% formic acid (FA). Resulting peptides were desalted using Sep-Pak SPE cartridge (Waters) according to manufactures instructions.

LC-MS/MS of tryptic peptides. LC-MS/MS analysis of digested peptides was performed on an Orbitrap Q Exactive Plus mass spectrometer (Thermo Fisher Scientific, Bremen) coupled to an EASY-nLC 1000 (Thermo Fisher Scientific). Peptides were loaded and separated at 250 nl.min^-1^ on a home-made C18 30 cm capillary column picotip silica emitter tip (75 μm diameter filled with 1.9 μm Reprosil-Pur Basic C18-HD resin, (Dr. Maisch GmbH, Ammerbuch-Entringen, Germany)) equilibrated in solvent A (0.1 % FA). Peptides were eluted using a gradient of solvent B (ACN ,0.1 % FA) using a 120 min long gradient. Mass spectra were acquired in data-dependent acquisition mode with the XCalibur 2.2 software (Thermo Fisher Scientific, Bremen) with automatic switching between MS and MS/MS scans using a top-10 method. MS spectra were acquired at a resolution of 70000 (at m/z 400) with a target value of 3 × 10^6^ ions. The scan range was limited from 300 to 1700 m/z. Peptide fragmentation was performed using higher-energy collision dissociation (HCD) with the energy set at 28 NCE. Intensity threshold for ions selection was set at 1 × 10^6^ ions with charge exclusion of z = 1 and z > 7. The MS/MS spectra were acquired at a resolution of 17500 (at m/z 400). Isolation window was set at 1.6 Th. Dynamic exclusion was employed within 45s.

**Data analysis**. Raw files were searched using MaxQuant (version 1.5.3.8) (with the Andromeda search engine) against a *Vibrio cholerae strain ATCC 39315* database (3871 entries, downloaded from Uniprot in June 2017). The following search parameters were applied: carbamidomethylation of cysteines was set as a fixed modification, oxidation of methionine and protein N-terminal acetylation were set as variable modifications. The mass tolerances in MS and MS/MS were set to 5 ppm and 20 ppm respectively. Maximum peptide charge was set to 7 and 7 amino acids were required as minimum peptide length. A false discovery rate of 1% was set up for both protein and peptide levels. The iBAQ algorithm of MaxQuant was used to rank proteins according to their abundance in each sample.

**Yeast two-hybrid assays** were performed with plasmids pAS2ΔΔ (bait, TRP+) and pACTIIst (prey, LEU+) and the diploid *Saccharomyces cerevisiae* strain Y187xCG1945 (*leu*^-^ *trp*^-^ *his*^-^) as developed in Fromont-Racine *et al* [2]. Prey and bait plasmids were transformed as described [3], using drop-out medium without leucine and tryptophan. Interaction was tested in drop-out medium without leucine, tryptophan and histidine, in the presence of 1 mM 3-aminotriazole. X-gal overlay assay was performed as described [2].

**Cruciform extrusion assays during integron recombination** were performed as described in Loot et al 2010 (ref [46] in manuscript).

**Supplementary references**

1. Park, J.S., M.T. Marr, and J.W. Roberts, *E. coli Transcription repair coupling factor (Mfd protein) rescues arrested complexes by promoting forward translocation.* Cell, 2002. **109**(6): p. 757-67.

2. Fromont-Racine, M., J.C. Rain, and P. Legrain, *Toward a functional analysis of the yeast genome through exhaustive two-hybrid screens.* Nat Genet, 1997. **16**(3): p. 277-82.

3. Gietz, R.D. and R.H. Schiestl, *High-efficiency yeast transformation using the LiAc/SS carrier DNA/PEG method.* Nat Protoc, 2007. **2**(1): p. 31-4.
