# Supplementary figures and images for: RadD Contributes to R-Loop Avoidance in Sub-MIC Tobramycin
Source: mBio. 2019 Jul 2;10(4):e01173-19. doi: 10.1128/mBio.01173-19 (PMC6606805; doi:10.1128/mBio.01173-19)

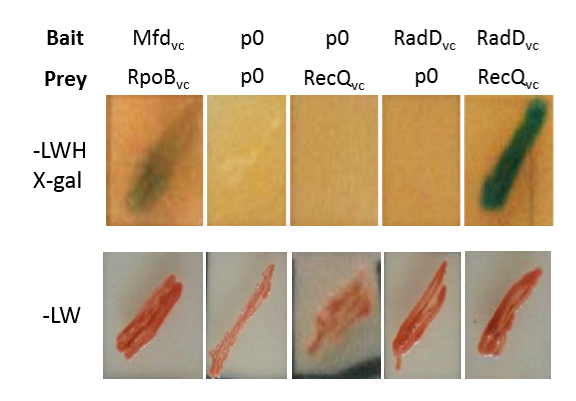

Supplement: FIG S2 [file mBio.01173-19-sf002.tif]
